# Novel Automated Suturing Technology for Minimally Invasive Mitral Chord Implantation: A Preclinical Evaluation Study

**DOI:** 10.1177/15569845221133381

**Published:** 2022-11-29

**Authors:** Paul Werner, Claus Rath, Christoph Gross, Niv Ad, Igor Gosev, Hossein Amirjamshidi, Thomas Poschner, Iuliana Coti, Marco Russo, Markus Mach, Alfred Kocher, Guenther Laufer, Jude Sauer, Martin Andreas

**Affiliations:** 1 Department of Cardiac Surgery, Medical University of Vienna, Austria; 2 Department of Surgery, University of Maryland School of Medicine, Baltimore, MD, USA; 3 Department of Surgery, Medical University of Rochester, NY, USA; 4 San Camillo Forlanini Hospital of Rome, Italy

**Keywords:** mitral regurgitation, mitral valve repair, chordal replacement, automated ePTFE suturing, new ePTFE titanium fastener

## Abstract

**Objective:**

This study evaluated the ergonomics and time requirements of using a novel automated suturing and titanium fastener deployment technology for chordal replacement in human heart specimens in open and minimally invasive cardiac surgery (MICS) simulators.

**Methods:**

Five cardiac surgeons used novel, manually powered expanded polytetrafluoroethylene (ePTFE) suturing devices to automate suture placement between mitral leaflets and papillary muscles in explanted cadaver hearts, along with customized titanium fastener delivery devices to secure suture and trim suture tails. This mitral chordal replacement test was conducted using surgical models simulating open and MICS mitral repair access. The study was approved by the institutional ethical board.

**Results:**

After a brief introduction to this technique using plastic models, study surgeons performed 48 chordal replacements in human mitral valves, placing 18 in an open model and 30 in a right minithoracotomy model. The time range to complete a single chordal replacement was between 55 s and 8 min, with an overall mean duration of 3.6 ± 1.5 min. No difference in duration of implantation was recorded for the MICS and open sternotomy simulators used. Good control of suture delivery was reported in 95.8% (*n* = 46) of leaflet aspect of the sutures and in 100% (*N* = 48) of papillary muscle sutures.

**Conclusions:**

Automated mitral chordal ePTFE suturing simulated through open and MICS access demonstrated quality handling and accurate placement of sutures in human heart specimens. A clinical trial using this technology is currently ongoing. This innovation may present an important advance facilitating enhanced minimally invasive mitral valve repair.

**Central Message**

Five cardiac surgeons evaluated automated ePTFE suture placement and securing technology for mitral chordal replacement in open and MICS simulator models. This cadaver heart study demonstrated potentially reduced procedure times while providing excellent ergonomics and reliable suture placement. This novel technology represents innovation toward enhancing minimally invasive mitral repair.

## Introduction

Surgical mitral valve repair (MVr) offers excellent results and is considered the gold standard for patients presenting with primary mitral regurgitation (MR).^1–3^ The “resect” (resection of excess tissue) and “respect” (chordal replacement without resection using expanded polytetrafluoroethylene [ePTFE] suture) concepts, used in combination with annuloplasty, have emerged as valuable approaches to treat mitral valve incompetence, with reported comparable outcomes in early and midterm follow-up. However, a growing body of evidence suggests that ePTFE chordal replacement offers improved long-term durability.^4–6^ Ex vivo studies have demonstrated better leaflet mobility and coaptation length with synthetic chordal replacement,^7^ and a clinical randomized controlled trial has further supported the benefits of chordal replacement.^5^ A meta-analysis comparing both methods concluded that chordal replacement may lead to superior freedom from valve-related reoperation and even to increased left ventricular function after surgery.^6^ Achieving reliable outcomes with chordal replacement requires well-controlled placement of appropriate-length ePTFE sutures between selected mitral leaflets and their corresponding papillary muscles. While some high-volume centers report excellent results using chordal replacement through a lateral minithoracotomy, minimally invasive cardiac surgery (MICS) approaches increase procedure complexity because of limited range of movement, more difficult working angles, and potentially reduced visualization.^5,8^

Despite its challenges, MICS MVr has already become the preferred approach for many surgeons treating patients with primary MR.^9^ In MICS MVr procedures, patients can avoid the complications associated with a median sternotomy, such as deep sternal wound infections. Cosmetic advantages and cost reduction due to shorter hospital stays are also potential benefits from this approach. MICS MVr is frequently associated with prolonged procedural times. Less experienced surgeons may be discouraged from using MICS approaches for complex chordal replacement procedures to avoid jeopardizing the excellent patient outcomes expected from more traditional MVr surgery.^10^

An unmet clinical need exists for novel technology that further simplifies MICS mitral chordal replacement by reducing procedural complexity and surgeon dexterity requirements while maintaining optimal procedural exposure and results.

## Methods

### Technology

This study’s technology for mitral chordal replacement includes the Mi-STITCH™ Device (LSI Solutions, Inc., Victor, NY, USA) for automated ePTFE suturing during chordal replacement and the Mi-KNOT™ Device (LSI Solutions, Inc.) for securing ePTFE chords with a customized titanium fastener. The design intent of this novel technique is to provide faster, less technically challenging, and more reliable mitral chordal replacement under remote access conditions. The Mi-STITCH™ and Mi-KNOT™ Device design functionality was confirmed through a battery of in vitro tests intended to simulate the clinical requirements of mitral chord replacement. This confirmation was substantiated by the implementation of a robust risk assessment and the extraordinary performance of the Mi-KNOT™ technology compared with each specification. In addition, a number of in vivo studies support the concept of the proposed device and the suitability of the study center for the proposed investigation, including a study of 8 sheep in a 6-month survivor recovery model following mitral valve replacement surgery using a developmental precursor to the proposed Mi-STITCH/Mi-KNOT Device system^11^ and a subsequent study of atrioventricular valve repair in live pigs,^12^ which further demonstrated competence and suitability of the proposed study elements.

**Fig. 1.**
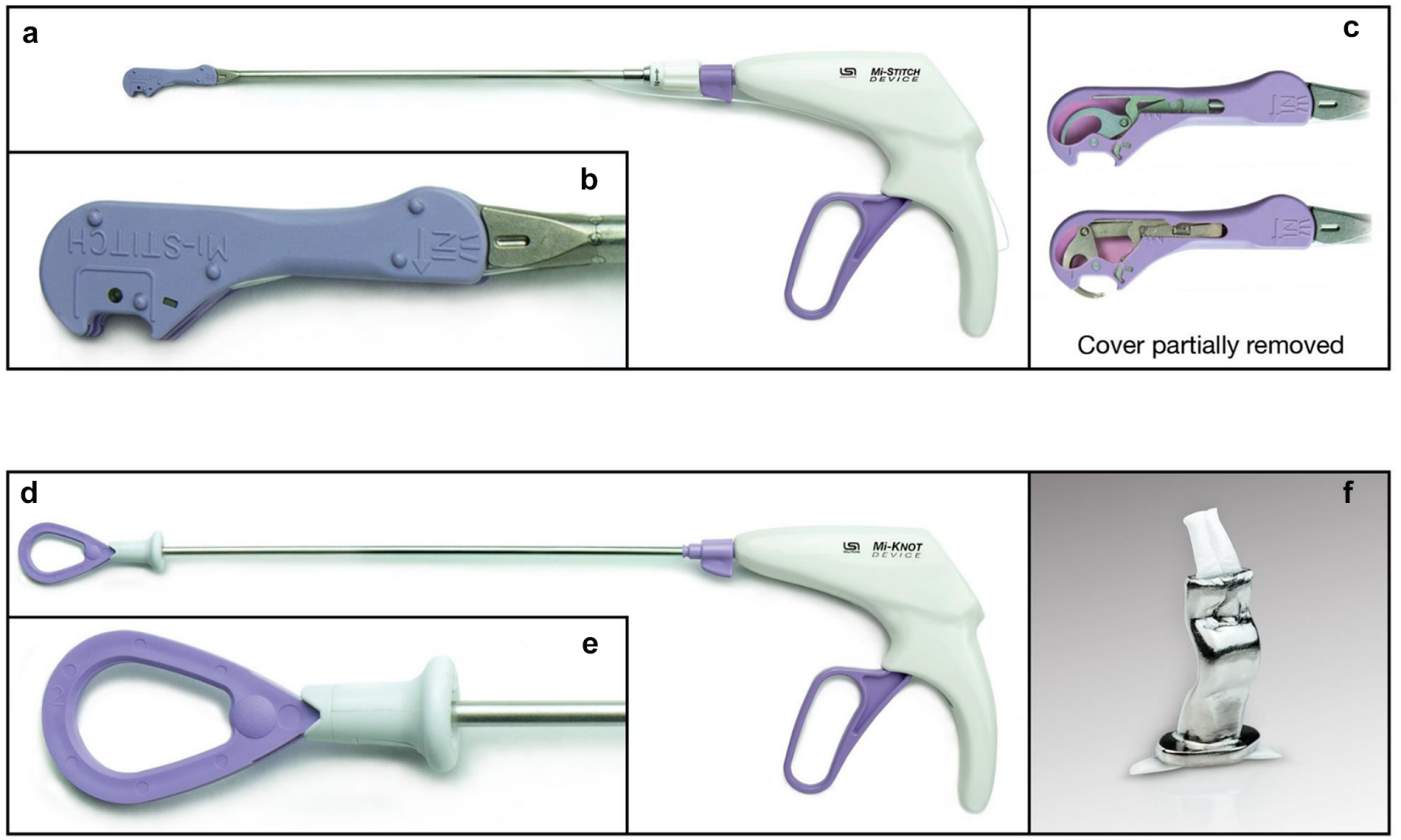
The Mi-CHORD™ system (LSI Solutions, Inc., Victor, NY, USA) for automated ePTFE suture delivery and securement during mitral valve chordal implantation. (a) The Mi-STITCH™ Device (LSI Solutions, Inc.) for automated suture delivery. (b) Close-up image of the Mi-STITCH™ Device tip. (c) The Mi-STITCH™ Device tip with cover partially removed to demonstrate internal mechanisms for needle deployment during lever squeeze. (d) The Mi-KNOT™ Device (LSI Solutions, Inc.) for reliably delivering a titanium fastener to secure ePTFE artificial chordae. (e) Close-up image of the Mi-KNOT™ Device tip. (f) Close-up image of a crimped Mi-KNOT™ titanium fastener securing ePTFE suture. ePTFE, expanded polytetrafluoroethylene.

Each sterile device kit includes both a Mi-STITCH™ Device and a Mi-KNOT™ Device. The Mi-STITCH™ Device (Fig. 1a) was provided preloaded with an LS-5™ ePTFE suture. This device’s handle and lever were connected to a shaft that provides 360° of rotation, with a distal tip that can be articulated ~15° in either direction. The distal tip features an open jaw for receiving tissue (Fig. 1b–c). For the first tissue bite in this technique, by squeezing the lever, a pair of curved needles integrated within the distal tip advanced through the targeted mitral leaflet tissue positioned within the jaw. Once through the tissue, the needles engaged distal suture endcaps attached to each end of the preloaded ePTFE suture. Releasing the lever retracted the needles, along with the now engaged ePTFE suture ends, back through the tissue in the jaw. With the jaw positioned away from any tissue, a second squeeze and release of the lever re-armed the device by transferring the suture and its endcaps back into their starting position at the distal end of the jaw, preparing the device for a second tissue bite. The surgeon next positioned the device tip jaw onto the base of the appropriate corresponding papillary muscle. A third squeeze and release of the lever created an untied ePTFE suture loop between the mitral leaflet and the papillary muscle. The Mi-STITCH™ Device and its suture ends were withdrawn back out through the access site. Per surgeon preference, pledgets could optionally be placed on either side of the mitral leaflet or papillary muscle.

With the ends of the suture now extracorporeal, the endcaps were cut away, and the Mi-KNOT™ Device (Fig. 1d–e) was used to deploy a custom titanium fastener for securing the ePTFE suture. The free suture ends were snared and pulled through the hollow diamond-shaped titanium fastener preloaded within the distal end of the device. The device tip with titanium fastener was slid down over the suture to near the base of the papillary muscle, where the suture loop was tightened to the precise chordal length preferred by the surgeon. With a single squeeze of the lever, the titanium fastener was crimped to securely hold the suture in place, and the redundant suture tails were automatically cut (Fig. 1f). Unlike the hand-tied knots currently used for traditional ePTFE suture chordal replacements, which are frequently left within or near the leaflet coaptation zone, the titanium fasteners used in this study were intentionally placed near the base of the papillary muscle, away from critical tissue structures. The device shaft could be rotated 360° to enable surgeons to orient suture tails away from native leaflets, chordae tendineae, and the coaptation zone.

### Study Design

Five board-certified senior heart surgeons participated in this research to assess the usability and effectiveness of the new devices. The study included the use of 20 explanted cadaver hearts to simulate both open and MICS MVr settings (Supplemental Video). The human heart specimens were harvested without chemical preservatives. All contributing donors consented to donate their bodies “for the training of doctors, for further medical education and for medical science.” The study was approved by the Medical University of Vienna ethical board (Ethical board No. 1229/2020, approval July 7, 2020).

**Fig. 2.**
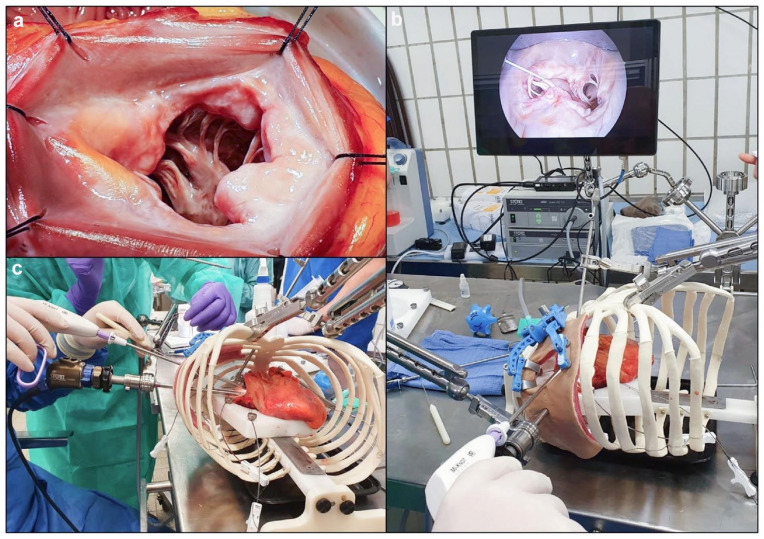
The customized high-fidelity cardiac surgery simulator used to simulate mitral valve synthetic chordal implantation procedures. (a) An image of the open exposure of the mitral valve subvalvular apparatus. (b) (c) Images of the minimally invasive access model with endoscopic visualization of the mitral valve.

Prior to suturing in the human heart specimens, all participating surgeons received a brief introduction to the study devices, which included each surgeon firing both devices once or twice in a plastic mitral valve model. The study objective and its open and MICS simulators (Fig. 2a–c) were then explained to the surgeons. In the open mitral repair model (Fig. 2a), human heart specimens were positioned on a customized cradle with the left atrium partially excised and the left ventricle supported to preserve the orifice shape. In the MICS simulator, human heart specimens were positioned within an anatomically accurate thoracic cavity frame incorporating a plastic spine and ribs with a 5 to 6 cm incision through an opaque compliant plastic membrane, representing lateral minithoracotomy access. Video imaging of the surgical field was provided with a 0° video endoscope (Storz, Baden-Württemberg, Germany; Fig. 2b–c). Each surgeon performed a maximum of 10 chordal replacements with the Mi-STITCH™ and Mi-KNOT™ Devices in both stations. Upon test completion, each surgeon was provided with a questionnaire to assess device ergonomics and function. This questionnaire was designed to evaluate the ease and effectiveness of suture and fastener delivery.

## Results

Of the 48 chordal replacements performed in human heart specimens by 5 senior surgeons in this study, 18 (37.5%) were performed in the open model and 30 (62.5%) in the MICS model. The surgeons selected the following cadaver mitral leaflet segments as targets: P1 in 1 (2.1%), P2 in 29 (60.4%), A1 in 1 (2.1%), A2 in 16 (33.3%), and A3 in 1 (2.1%) chordal placement. During leaflet suturing, surgeons reported acceptable control in 46 of 48 placements (95.8%). One surgeon, in his first 2 consecutive chordal replacements in the open model, reported delivering the suture bite too deep in the targeted P2 leaflet. All surgeons reported excellent ability to control the location of suture delivery during papillary suturing for every bite (*N* = 48, 100%). The Mi-STITCH™ Device was successfully rearmed with no reported difficulties with any test. Study surgeons reported satisfaction with the adjustability of chords to the desired length in 44 chordal placements, whereas 4 length reports were not captured. The mean duration to complete chordal replacement across all 48 tests was 3.6 ± 1.5 min (range, 55 s to 8 min). There was no significant difference in the mean duration in the open (3.39 ± 1.27 min) versus MICS (3.67 ± 1.63 min) models (*P* = 0.44). While all participants reported similarly favorable satisfaction with this technique, individual surgeons demonstrated noticeable variability in the time required to complete each test step. For example, in the MICS simulator model, time to perform each required technique step in this study ranged from 1.76 min for the fastest surgeon to 4.12 min for the slowest surgeon.

## Discussion

Successful surgical placement of ePTFE sutures as replacement mitral chords to enhance mitral leaflet coaptation can enable anatomical restoration of native mitral valve competency, with proven excellent long-term results in patients with degenerative mitral disease.^1,2^ Minimally invasive MVr with ePTFE chordal replacement through small access sites limits range of motion and impedes direct vision. The purpose of this study was to evaluate the ergonomics and time requirements of 5 senior cardiac surgeons using a novel automated suturing and titanium fastener deployment technology for chordal replacement in human heart specimens in open and MICS simulators. This preclinical evaluation demonstrated that after only a brief introduction, heart surgeons can use the Mi-STITCH^™^ Device to place ePTFE chords and the Mi-KNOT^™^ Device to deploy titanium fasteners under realistic simulator conditions with short procedure times and excellent control, accuracy, and ergonomics.

The use of the ePTFE suture for mitral chordal replacement is an established technique used by many mitral surgeons to reestablish mitral leaflet coaptation and valve competency with excellent long-term results in patients with degenerative MR.^13^ In recent years, mitral valve surgery has increasingly become a minimally invasive procedure. Alternative techniques to address the recognized suturing and knot-tying challenges related to ePTFE suture use, especially through small access wounds, have been proposed, with various subsequent levels of adoption. In 1999, for example, the “loop technique” using multiple preassembled loops of ePTFE suture at determined lengths has seen significant clinical adoption with excellent midterm results comparable with proven techniques, including leaflet resection.^4,14^ However, remote access MVr through smaller, less traumatic incisions remains a technically challenging and time-intensive operation for many surgeons. Many patients, especially those with more complex mitral disease, such as large or multiple-segment prolapse and anterior leaflet involvement, are often denied the many potential healing benefits offered by a less invasive surgical option.

The technology evaluated in this study is intended to reduce the technical challenges of accurate ePTFE suture placement in routine and complex MVr surgery, even under less invasive conditions. Despite the intrinsic challenges associated with minimally invasive access in this simulator, the mean duration for suture placement was less than 4 min and not significantly different from the open model. Technology and techniques to enable reliable, readily achieved, and accurate ePTFE chordal replacement may reduce cross-clamp and cardiopulmonary bypass times to increase the availability of MICS MVr to more patients.

A broadly accepted, easily implemented, and reliable technique to secure ePTFE suture in MVr remains elusive. The inconsistencies of hand-tying knots in slippery ePTFE suture can result in the tether between the leaflet and papillary muscle becoming too short or too long during tying or can allow the knots to untie. While several methods have been described to assess and establish effective replacement ePTFE suture chordal length,^15,16^ there is an unmet clinical need for a very reliable, easily performed option, especially in less invasive surgery.

**Fig. 3.**
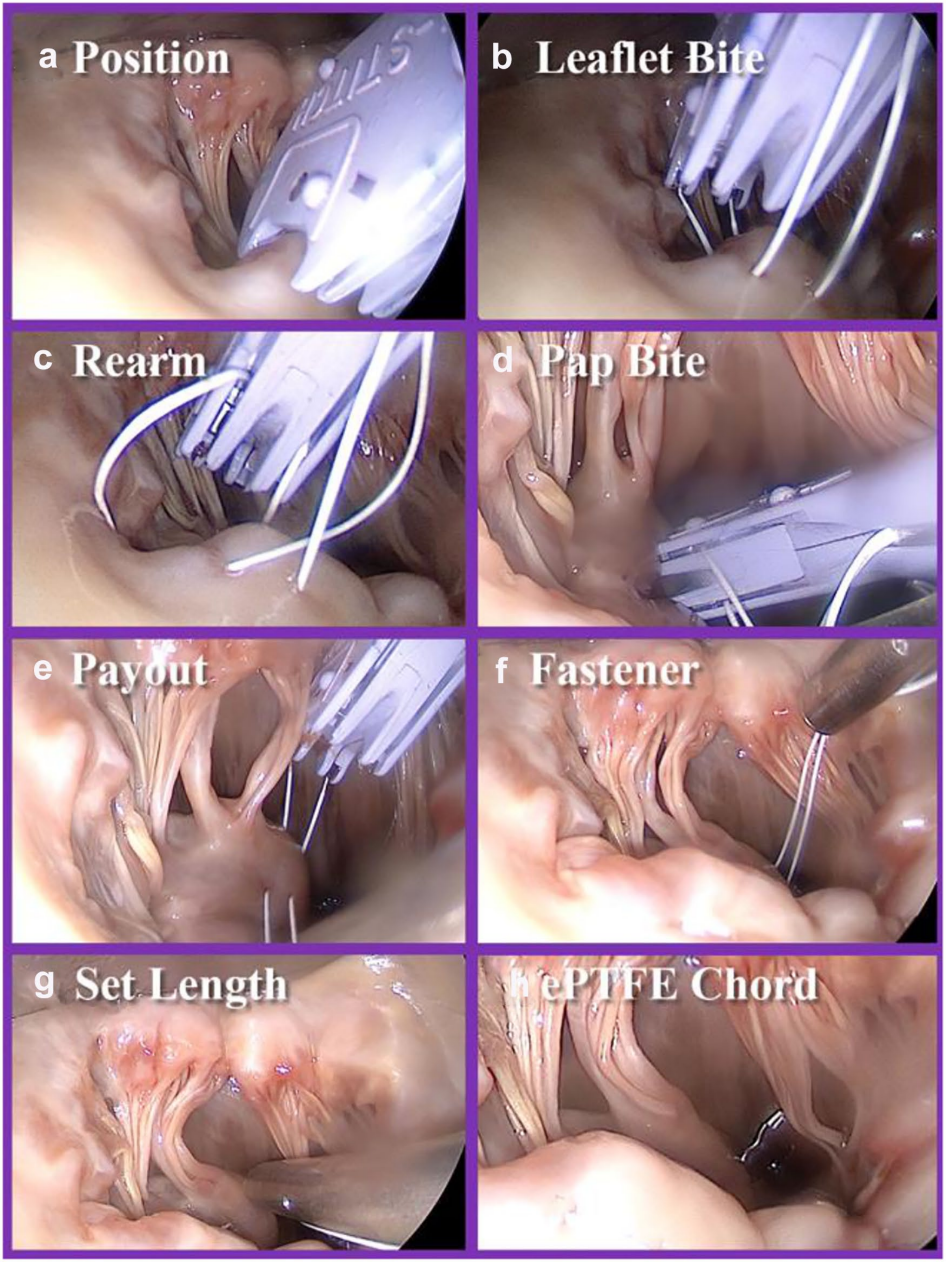
Procedure steps for chordal implantation with endoscopic visualization, as used in this study. The procedure begins with (a) positioning of the Mi-STITCH™ Device (LSI Solutions, Inc., Victor, NY, USA) and (b) placing the leaflet bite on the appropriate position of the mitral leaflet. The device is then (c) rearmed and (d) the papillary bite is taken and the (e) suture pays out of the device. (f) The ePTFE suture is then snared within the Mi-KNOT™ Device (LSI Solutions, Inc.), and the tip is brought into the surgical field. (g) Length customization of the synthetic chord is performed per surgeon preference, and the (h) titanium fastener is then deployed to secure suture, forming the full synthetic ePTFE chord.

The Mi-KNOT™ Device delivers a very strong mechanical ePTFE suture fastener through remote surgical sites with a single squeeze of its lever and automatically trims away excess suture tails. The reliable deployment of a durable titanium fastener allows the chordal replacement ePTFE suture loop to remain at the exact length set by the surgeon. In addition, this titanium fastener is positioned near the base of the papillary muscle instead of near the coapting leaflets or other delicate tissue structures (Fig. 3h).

### Future Outlook

Following extensive preclinical development and testing, including the described ex vivo experiment, the Austrian competent authority approved a first-in-human study using the Mi-STITCH™ and Mi-KNOT™ Devices. A phase 1 trial was initiated, and 12 patients successfully underwent MVr procedures using these devices; clinical follow-up is ongoing. A dedicated phase 2 trial with enrollment of a larger cohort is planned to start by the end of 2022.

## Conclusions

The evaluated technology represents promising innovation in the field of minimally invasive MVr using ePTFE suture for mitral chordal replacement. The automated suturing device and titanium fastener placement device were demonstrated by 5 practicing heart surgeons to readily provide acceptable chordal replacement results in human heart specimens through open and MICS MVr simulators. Supportive preclinical study results, along with the initial early clinical experience, encourage further exploration of this new approach to enhancing ePTFE suture placement for open and especially MICS mitral valve chordal replacement.

## Supplemental Material

Visual abstract – Supplemental material for Novel Automated Suturing Technology for Minimally Invasive Mitral Chord Implantation: A Preclinical Evaluation Study Click here for additional data file. Supplemental material, sj-pptx-1-inv-10.1177_15569845221133381 for Novel Automated Suturing Technology for Minimally Invasive Mitral Chord Implantation: A Preclinical Evaluation Study by Paul Werner, Claus Rath, Christoph Gross, Niv Ad, Igor Gosev, Hossein Amirjamshidi, Thomas Poschner, Iuliana Coti, Marco Russo, Markus Mach, Alfred Kocher, Guenther Laufer, Jude Sauer and Martin Andreas in Innovations: Technology and Techniques in Cardiothoracic and Vascular Surgery
