# Supplementary material for: Novel Automated Suturing Technology for Minimally Invasive Mitral Chord Implantation: A Preclinical Evaluation Study
Source: Innovations (Phila). 2022 Nov 29;17(6):506–12. doi: 10.1177/15569845221133381 (PMC9846373; doi:10.1177/15569845221133381)
Supplement: Visual abstract – Supplemental material for Novel Automated Suturing Technology for Minimally Invasive Mitral Chord Implantation: A Preclinical Evaluation Study [file sj-pptx-1-inv-10.1177_15569845221133381.pptx]

## Slide 1
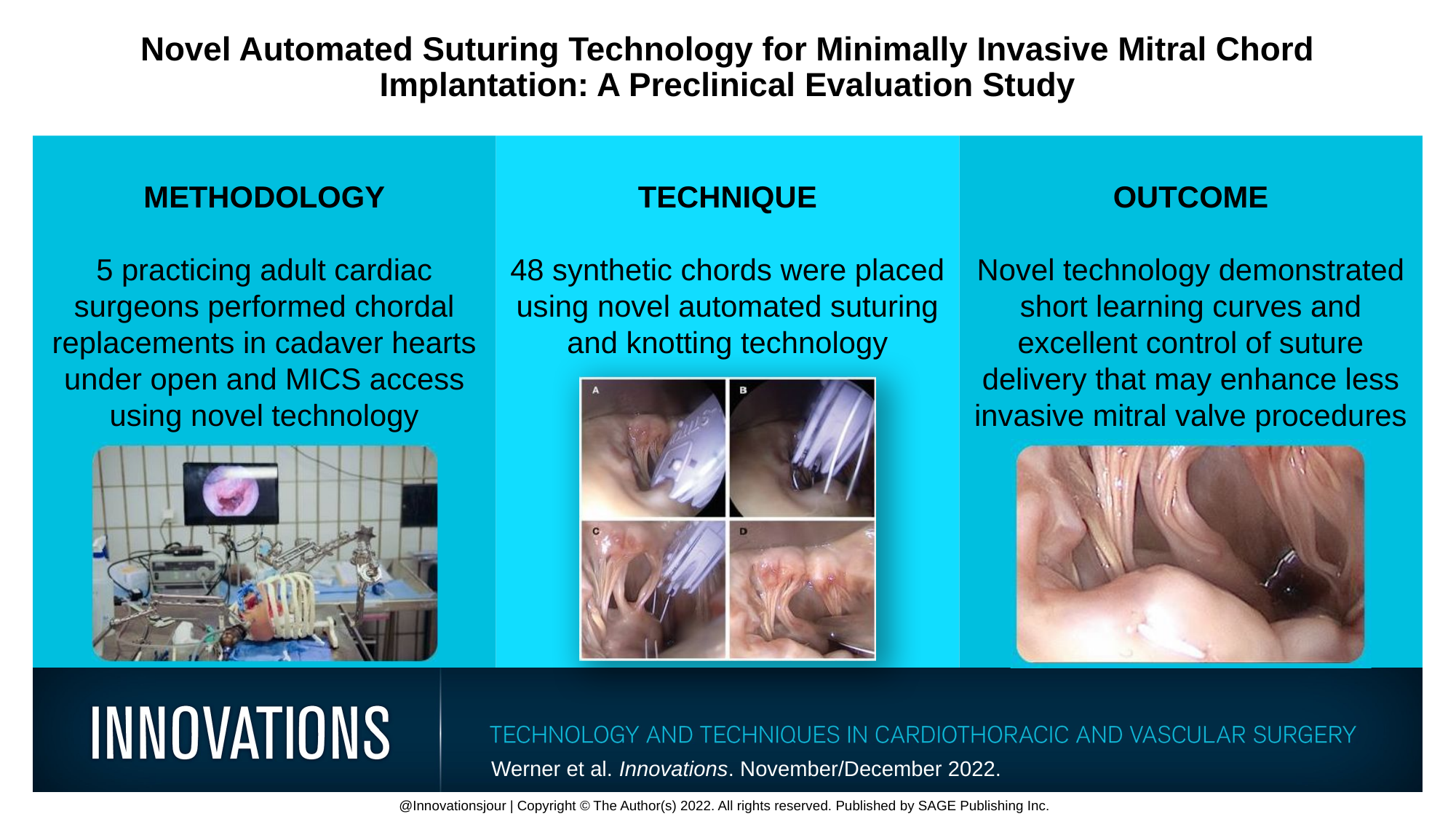

# Novel Automated Suturing Technology for Minimally Invasive Mitral Chord Implantation: A Preclinical Evaluation Study
METHODOLOGY
5 practicing adult cardiac surgeons performed chordal replacements in cadaver hearts under open and MICS access using novel technology
TECHNIQUE
48 synthetic chords were placed using novel automated suturing and knotting technology
OUTCOME
Novel technology demonstrated short learning curves and excellent control of suture delivery that may enhance less invasive mitral valve procedures
Werner et al. Innovations. November/December 2022.
@Innovationsjour | Copyright © The Author(s) 2022. All rights reserved. Published by SAGE Publishing Inc.
